# Mortality, Risk Factors and Risk Assessment after Periprosthetic Femoral Fractures—A Retrospective Cohort Study

**DOI:** 10.3390/jcm10194324

**Published:** 2021-09-23

**Authors:** Thaqif El Khassawna, Gero Knapp, Nadja Scheibler, Deeksha Malhan, Nike Walter, Christoph Biehl, Volker Alt, Christian Heiss, Markus Rupp

**Affiliations:** 1 Experimental Trauma Surgery, Faculty of Medicine, Justus-Liebig-University, 35392 Giessen, Germany; thaqif.elkhassawna@chiru.med.uni-giessen.de (T.E.K.); n.scheibler@gmx.de (N.S.); deeksha.malhan@charite.de (D.M.); christian.heiss@chiru.med.uni-giessen.de (C.H.); 2 Department of Trauma, Hand and Reconstructive Surgery, University Hospital, 35390 Giessen, Germany; gero.knapp@chiru.med.uni-giessen.de (G.K.); Christoph.Biehl@chiru.med.uni-giessen.de (C.B.); volker.alt@ukr.de (V.A.); 3 Institute for Theoretical Biology (ITB), Charité—Universitätsmedizin, Corporate Member of Freie Universität Berlin, Humboldt—Universität zu Berlin and Berlin Institute of Health, 10117 Berlin, Germany; 4 Molecular Cancer Research Center (MKFZ), Medical Department of Hematology, Oncology and Tumor Immunology, Charité—Universitätsmedizin Berlin, Corporate Member of Freie Universität Berlin Humboldt—Universität zu Berlin and Berlin Institute of Health, 10117 Berlin, Germany; 5 Department of Trauma Surgery, University Hospital, 93053 Regensburg, Germany; nike.walter@ukr.de

**Keywords:** periprosthetic fracture, periprosthetic femur fracture, geriatric trauma, mortality, Charlson comorbidity index

## Abstract

Periprosthetic femoral fracture (PFF) is a devastating complication. Here, the authors aimed to determine the influence of the timing of surgery as a risk factor for mortality and poor postoperative outcome in patients suffering from PFF. A retrospective descriptive analysis of patients treated for PFF between January 2010 and March 2018 was performed. In addition to patient and treatment characteristics, we assessed mortality rates and postoperative functional outcome by using the Harris Hip and WOMAC score. One-year mortality after PFF was 10.7%. Delayed surgery after 48 h did not negatively influence mortality after PFF. The postoperative hospital stay did not influence the mortality rate, nor did it correlate with medical scores of comorbidities, general health or functionalities. Cementation of stem correlated negatively with the WOMAC score. Deceased patients had a higher Charlson Comorbidity Index (CCI) score, while American society of Anaesthesiologists (ASA) scores did not show a significant difference. There were no differences between ORIF and revision arthroplasty. In conclusion, delayed surgery after 48 h does not negatively influence mortality after PFF. The CCI seems to be a suitable tool to assess patients’ risk for increased mortality after PFF, while the usually used ASA score is not able to achieve a relevant risk assessment.

## 1. Introduction

Periprosthetic femoral fractures (PFF) represent an increasing challenge in orthopedic surgery. Due to the continuing increase in the number of total endoprostheses, a further increase in the incidence of patients with periprosthetic femur fractures is to be expected. Although it is a rare disease with a current incidence between 1% and 2.3% depending on the source, PFF is a tremendous challenge for both the surgeon and the patient [1,2]. The surgical outcomes of joint replacement surgery and healthcare are increasingly improving. Therefore, the procedure is performed more frequently on younger, more active patients as well as older multimorbid patients [3]. The risks for PFF are multifactorial, ranging from patient-specific risk factors (such as reduced bone quality, tendency to fall) and the technical execution of the endoprosthetic surgery (as femoral notching, small stems, uncemented implantation technique, and false drilling holes) [4]. The mortality rate after PFF is up to 10% in the first 30 days; Bhattacharyya and colleagues showed a rate of 11% mortality in the first year after surgical treatment [5,6]. The surgical treatment of PFF strictly follows the classification. In the case of a fixed femoral stem with osteosynthesis, the treatment with open reduction and internal fixation (ORIF) can preserve the prosthesis [7], whereas the ORIF results in a significantly increased failure rate when treating fractures around a loose stem [8,9]. Although ORIF is considered less radical than revision surgery, the impact of the treatment procedure on mortality/morbidity is not well reported. Reasons for the increased mortality rate can be explained by the old age of patients and their multimorbidities. In comparison to elective interventions, risk minimization by adequate preparation time and estimation can be very difficult in the treatment of PFF. Therefore, risk scores for a variety of acute and chronic diseases have been developed. The Charlson comorbidity index (CCI) has been validated for estimating long-term prognosis in the presence of multiple pre-existing comorbidities. A correlation between a high CCI and outcomes after surgery is controversial in the literature [10,11].

The aim of the present study was to descriptively assess the influence of the timing of surgery on mortality and postoperative outcome after PFF. Does a longer time between hospitalisation and surgery lead to a higher postoperative mortality rate or a worse physical outcome? Furthermore, we evaluated whether the American Society of Anaesthesiologists (ASA) score and the Charlson Comorbidity Index (CCI) provide an adequate tool to assess mortality risk in often severely morbid and elderly patients who underwent surgical treatment for PFF.

## 2. Materials and Methods

The study was approved by the institutional review board of the University Hospital Giessen and Marburg, Campus Giessen in Germany and was filed under the number AZ 184/17 on 20 December 2017. All included patients suffered from PFF after THA and were treated between January 2010 and February 2018 at the Department for Trauma, Hand and Reconstructive Surgery of the University Hospital Giessen and Marburg, Campus Giessen, a level-one trauma centre. The international classification of disease (ICD) codes S72, M96.6 and T84 of the German ICD-10 were used to identify the included patient as there is no specific code existing for PFF.

Patient records were analysed to collect information regarding the patient’s demography, hospitalisation, and details of surgery. The following parameters were included: age, reason and date of fracture and fracture classification by the Unified Classification System (UCS) [12]. To identify relevant correlations with treatment outcome, the length of hospital stays, and time from primary implantation to PFF were recorded. The time from admittance in hospital to surgery was grouped into surgery within 48 h and surgery after 48 h, since 48 h could be shown to be a relevant time frame for lower mortality and morbidity when surgery can be achieved within proximal femur fractures [13]. To evaluate comorbidity scores regarding outcome in PFF, the American Society of Anaesthesiologists (ASA) score and the Charlson Comorbidity Index (CCI) were assessed. The type of surgery was differentiated as prosthesis-retaining treatment, which included ORIF procedures or revision arthroplasty.

**Figure 1:**
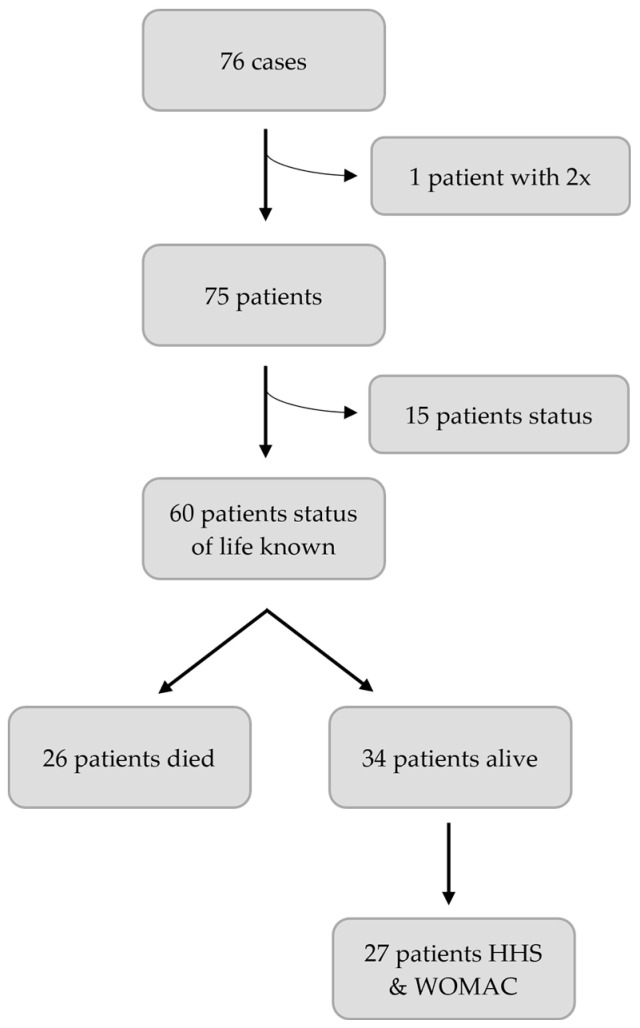
Flowchart study design.

For follow-up, patients were invited to our outpatient clinic. Harris Hip Score (HHS) and Western Ontario and McMaster Universities Osteoarthritis Index (WOMAC) were assessed. Patients who were not able to visit our hospital completed the questionnaires by telephone or by post. For deceased patients, relatives and general practitioners were contacted to determine the time of death. Figure 1 shows the study design.

Data were analysed using SPSS statistics version 27.0 (IBM, SPSS Inc., Armonk, NY, USA). Continuous parameters were presented as (Minimum: Maximum; Mean ± SD). Frequencies for all non-union risk factors were calculated. For analyses of the differences between patients, the chi-squared test or Fischer’s exact test were applied for categorical variables. Wilcoxon’s signed rank test and the Mann–Whitney U-test were applied for between-group comparisons. The critical value for significance was set at *p* ≤ 0.05. Data were presented in graphs as means. Whiskers in the bar graphs of patients counts show a 95% Confidence Interval (95% CI), whereas all other bar graphs show whiskers of the standard error of mean (±SEM).

Bivariate correlations were examined and shown in scatterplots. Pearson correlation coefficients were calculated for scale variables, while Kendall’s Tau-b was used for ordinal variables, and the two-tailed test of significance has a cut-off of *p* ≤ 0.05.

## 3. Results

**Figure 2:**
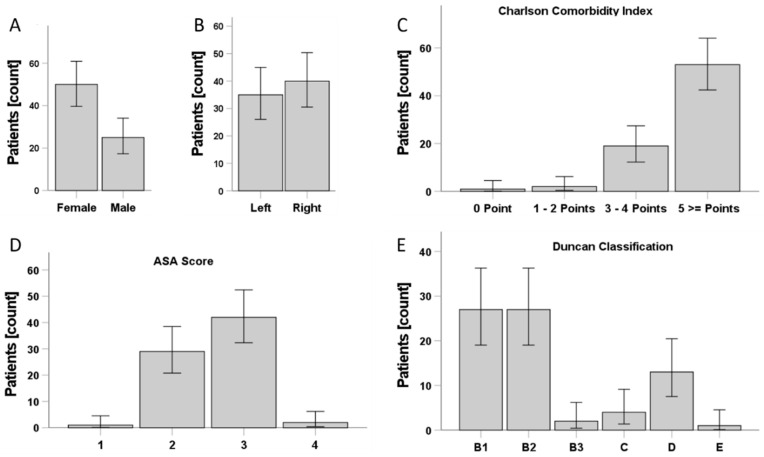
An overview of the patient cohort, their general condition and the periprosthetic fracture classification. (**A**) Gender of patients, (**B**) site of injury, (**C**,**D**) frequency and severity of CCI score among patients. (**E**) UCS classification.

Seventy-five patients with a mean age of 79.5 years (range 45.75–97.67; SD ± 8.31) and of both genders (Figure 2A) were included in the study. The ratio between the operated sides was nearly equal (Figure 2B). The general condition of the patients was determined preoperatively, using the CCI and the ASA score (Figure 2C,D). The CCI showed that patients had at least one comorbidity, 29.3% of patients had less than five comorbidities, whereas the majority, with 70.7%, had five or more comorbidities (range 1–16; mean 6.28 ± 2.79 SD).

The ASA score showed that only 1.3% of all patients did not suffer systemic diseases (ASA I), while the majority of the patients had severe systemic diseases (56% ASA III) (Figure 1D). The UCS classification showed that most patients were classified as B1 and B2 with 36% each (Figure 2E). An overview of the patient population is given Table 1.

The reason for the first endoprosthesis implantation in our collective was mainly the presence of primary osteoarthritis. Post-traumatic conditions or infections had a minor role (Table 2).

The time between primary implantation of the endoprostheses and the occurrence of PFF showed a vast range of 0 to 439 months (mean 121.26 ± 97.93 SD). The mortality rate 1 year after surgery was 66% in the entire cohort. Total mortality until the time of the last follow-up was 31 ± 27.12 months (range 0–87).

**Figure 3:**
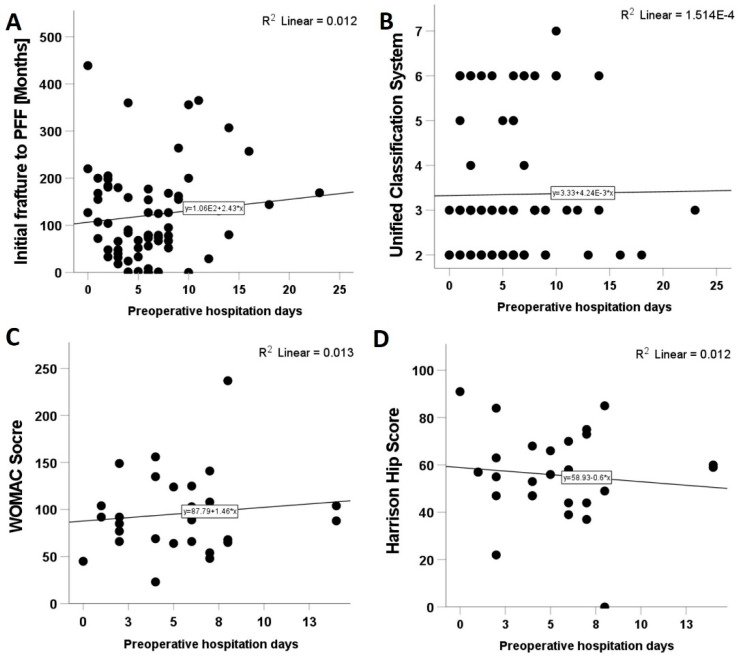
Correlation of preoperative hospitalization time with (**A**) initial time between initial fracture to PFF, (**B**) UCS severity, (**C**) the WOMAC score and (**D**) the Harris Hip score.

The average time of hospitalization was, in days (2:56; 20.21: ±9.7), with no statistically significant correlation to ASA or CCI. The HHS postoperatively at follow-up was (0:91; 55.78: ±19.166), with a poor outcome (HHS < 70) in 77.8% of patients. The WOMAC score had (23:237; 95.44: ±43.72) points. The current recommendation for surgical care in PFF is within the first 48 h after trauma. However, only 21 patients (28%), received surgery within the first 48 h, whereas other patients were operated on at later time points: 12 patients (16%) within 3–4 days, 16 patients (21.3%) within 5–6 days, and 26 patients (34.7%) were operated on after more than 7 days, (0: 23; 5.73: ±4.38). Comparing the groups, no statistical significance was found in favour of the earlier operation in terms of mortality rate. Furthermore, the preoperative days of hospitalization showed a positive correlation with the time between initial fracture (time point of the first implantation) and PFF, and with the UCS severity and with the WOMAC score (Figure 3A–C). The HHS correlation to the preoperative hospitalization days was negative (Figure 3D). However, none of these were statistically significant. Considering the type of surgical treatment, 46 patients (61.3%) underwent preservation of the prosthesis by ORIF, and 29 patients (38.7%) underwent revision arthroplasty. Likewise, there was no statistically significant difference between the type of surgical treatment and the mortality rate. Age also showed no effect on increased mortality. We also compared the mortality rate between ASA II and ASAIII patients; no statistically significant difference could be found (*p* = 0.304).

**Figure 4:**
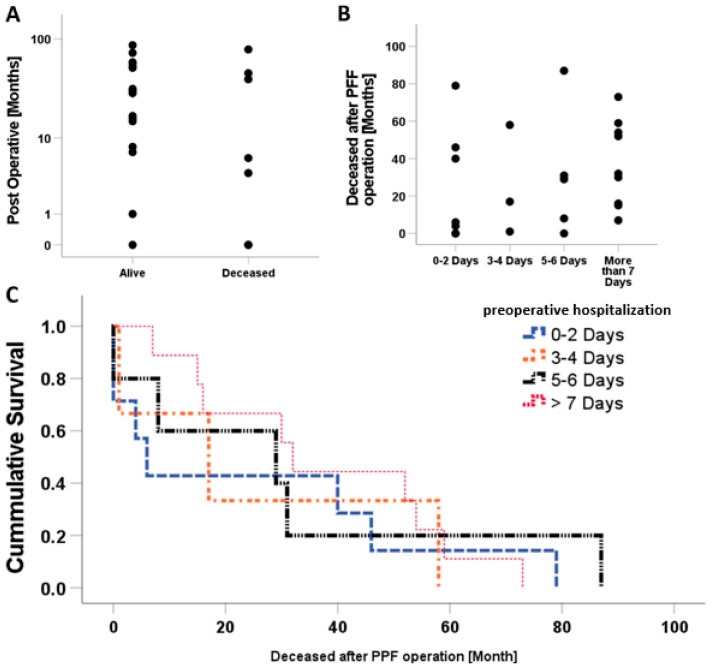
Correlation between mortality and time. (**A**) No statistically significant correlation between the numbers of patients survived or deceased after the operation. (**B**) Patient’s mortality was not affected by the duration of hospital stay pre-operatively. (**C**) Survival is not correlated to preoperative hospitalization days.

Mortality risk is one of the most counted results in periprosthetic fractures studies. However, no statistical significance was seen comparing the number of alive or deceased patients in correlation with their survival time postoperatively. Time to death was either directly postoperatively or ranged up to 87 months (0:87; 31 ± 27.1) (Figure 4A). One important aspect is the recommendation of surgical treatment within 2 days of trauma. Nonetheless, the data reflected no statistically significant difference when correlating the comparing number of deceased patients according to the time of preoperative hospitalization (Figure 4B). The cumulative survival was expressed as a Kaplan–Meier curve. The horizontal lines represent the duration of follow-up times occurred, inspected and when patients were still alive. A vertical drop in a Kaplan–Meier curve indicates an event; the horizontal drops here represent the time of death of patients postoperatively. Deaths very shortly after operation were seen in all groups apart from the group with more than 7 days of preoperative hospitalization. The first curve defined the survival depending on a preoperative hospitalization of less than 2 days (blue line). The longest duration is seen in the group of 5–6 days, followed by the group of 0–2 days. In other words, the follow up in longer intervals was for 5–6 days of preoperative hospitalization implants, and then the 0–2 days (Figure 4C).

## 4. Discussion

The treatment algorithm for periprosthetic femoral fractures has been continuously developed in recent years and is largely standardized [4,14,15]. The challenging task in the coming years will be to adequately provide care for the ever-increasing number of patients [16]. In addition to improving the quality of surgical treatment, risk management and the control of complications are essential [13,17,18,19]. The present retrospective study specifically investigated the postoperative mortality risk after PFF. The aim was to identify possible risk factors in order to ideally recognize and minimize them preoperatively.

**Figure A1:**
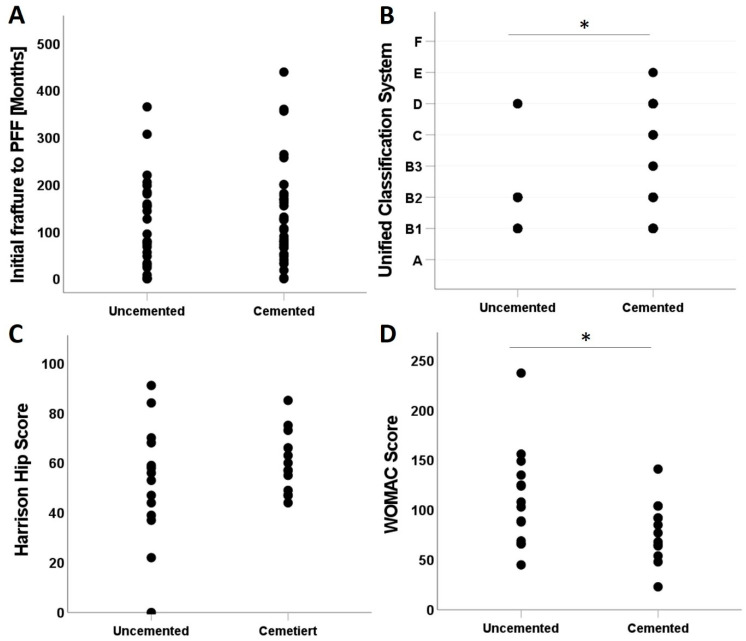
Impact of cementation on (**A**) initial time between initial fracture to PFF, (**B**) UCS severity, (**C**) the Harris Hip score, and (**D**) the WOMAC score. *: p-Value <= 0.05.

The mortality rate was recorded in eight patients (10.7%) within 1 year, including three inpatient deaths (4%), which is comparable to previous findings [9,17,20,21]. Our study has some limitations that are important to mention. We were not able to include all 75 patients in the follow-up. In a monocentre study, the availability of patient-level data is an advantage compared with a national registry. However, our sample size was too small to achieve statistical significance despite clinically significant trends. There are factors that affect the calculation of power that were not considered in our study, such as accounting for confounding factors such as cementation of the stem (Figure A1). This would require an increase in the size of the sample covered; however, this is only possible through registry data or a multicentre study. This, however, will make the acquisition of WOMAC and CCI scores very hard in a large sample.

However, our study includes two unique scribers, the use of a non-orthopaedic score to estimate mortality risk and the in-hospital experience of the relevance of preoperative waiting time. However, further studies with adequate sample sizes are required to assert these finding.

As with all retrospective analyses, one must be cautious about overinterpreting the associations found here as causal. Elderly patients often have multiple comorbidities that increase the intraoperative and postoperative mortality risk. For these reasons, identification of preoperative risk factors is essential for all surgeries in general. Summarizing comorbidities using a score is helpful to fully understand the patient’s general condition. While the ASA classification has been shown to be too inaccurate as a prevalence factor in surgery, the CCI seems to provide a better assessment for postoperative mortality risk here [10,11]. In this cohort, we confirmed this with a significant trend. Elevated CCI was associated with an increased risk of mortality where, for example, in the ASA classification there was no difference between class 2 and class 3 in terms of mortality. However, in such a cohort regarding age and comorbidities, the percentage of ASA I patients is very low, and the mortality of patients with an ASA IV score is usually high. A large number of studies have proclaimed the importance of early surgical treatment in PFF [5,22,23]. Only 28% of patients in our collective underwent surgery within the first 48 h after trauma, whereas the majority of patients received surgery at a later time point. However, we did not find any difference in mortality between these groups. A possible interpretation of this difference to former studies is the time span between the former and present studies. Meanwhile, awareness of need in patient optimization and geriatric assessment have evolved ever since. Continuous improvement in both preoperative and postoperative care for geriatric patients can help further minimize risks [24]. Whether this is the reason for the lack of influence of the timing of surgery on the mortality rate in our cohort can only be conjectured and needs further investigation. Recently published data from a German geriatric trauma registry analysing timing of surgery do not show any influence on early and midterm mortality (120 days follow-up after PFF surgery), which is in line with our findings [25]. Differences to older studies might be due to the same reasons as outlined above. A similar trend was seen with the outcome in the functional scores, with timing of surgical care not affecting either a positive or negative score in the HHS or WOMAC score. Another aspect we investigated was the outcome and mortality depending on the initial type of fixation as cemented or uncemented stem. We could not detect any influence on the survival rate of the initial prosthesis until PFF. Solely the group with primary cemented stems performed better in the WOMAC score. One reason for this can certainly be found in the heterogeneity in fractures with a higher number of UCS C, D and E fractures.

In the choice of surgical treatment, the preservation of the prosthesis by means of open reduction and internal fixation is usually considered to be less stressful for the patient. Interestingly, despite a high mean CCI in our cohort, the type of surgical care did not influence increased mortality. Obvious factors influencing an increased mortality risk after surgical treatment of PFF could not be unambiguously identified. Many factors contributing to this increased mortality often cannot be influenced. However, it is important to assess preoperatively all risks that can be minimized and to treat them preventively.

## 5. Conclusions

There were no obvious factors influencing the mortality risk of PFF. In particular, we could not confirm the still-valid recommendation for prompt surgical treatment within the first 48 h after trauma. Therefore, the time prior to surgery should be used preoperatively to correctly assess and, if necessary, optimize the individual surgical risk of each patient. Internal medicine and anaesthesiology scores such as the Charlson Comorbidity Index or ASA classification provide some assessment of postoperative mortality risk in PFF. However, an individual risk assessment should be performed when treating PFF in a geriatric multimorbid patient population.

## Figures and Tables

**Table 1:** General patient population.

**Age**	79.5 Years	(45.75–97.67; SD ± 8.31)		
**Gender**	females	66.7%	males	33.3%
**Operated side**	left	46.7%	right	53.3%
**ASA score**	ASA I	1.3%		
	ASA II	38.7%		
	ASA III	56%		
	ASA IV	2.7%		
**UCS classification**	B1	36%		
	B2	36%		
	B3	2.7%		
	C	5.3%		
	D	10.7%		
	E	1.3%		

**Table 2:** Reasons for first implantation.

Aetiology	Frequency	Percent
unknown	10	13.3
trauma	11	14.7
osteoarthritis	51	68.0
osteonecrosis	2	2.7
rheumatic arthritis	1	1.3
total	75	100.0

## Data Availability

The datasets generated and analysed in the current study are available from the corresponding author on reasonable request.

## Appendix A

The relationship between stem type, whether cemented or uncemented, with medical scores and time of initial fracture (time point of the first implantation) was also examined. However, cemented and uncemented stems did not correlate significantly with the time between initial fracture and the PFF (Figure A1A). The UCS showed a higher positive correlation in cemented stem when compared to the positive correlation with uncemented ones (Figure A1A, *p* = 0.040). HHS comparison between uncemented (0:91; 51 ± 23.72) and cemented (44:85; 59.85 ± 12.32) was not statistically significant. However, cemented stems reflected better outcomes with higher minimum and mean than uncemented and a lower standard deviation (Figure A1C).

Intriguingly, the WOMAC score showed a higher negative correlation in uncemented stems compared to cemented ones (Figure A1D, *p* = 0.23).
